# Systematic review and meta-analysis on the prevalence and risk factors of oral frailty among older adults

**DOI:** 10.3389/fmed.2025.1512927

**Published:** 2025-01-22

**Authors:** Pingping Huang, Linjing Wu, Rongxiang Zhang, Shiqi Chen, Yu Zhang, Yuan Chen

**Affiliations:** ^1^Department of Nursing, Xiamen Cardiovascular Hospital, Xiamen University, Xiamen, China; ^2^School of Nursing, Fujian University of Traditional Chinese Medicine, Fuzhou, China

**Keywords:** oral frailty, prevalence, risk factor, older adults, meta-analysis

## Abstract

**Objectives:**

The present study aim to systematically review the prevalence and influencing factors of oral frailty in older people.

**Methods:**

A search strategy was developed and implemented to systematically review literature across PubMed, Embase, Web of Science, MEDLINE (via EBSCOhost), CINAHL, the Cochrane Library, Scopus, China Knowledge Resource Integrated Database (CNKI), Wanfang Data, Chinese Biomedical Database (CBM), and Weipu Database (VIP), in accordance with the PRISMA 2020 guidelines. Our search encompassed studies published up to June 28, 2024, that investigated the prevalence or risk factors of oral frailty among older adults. Literature screening, data extraction, and quality assessment were independently performed by two researchers, followed by data analysis using Stata 17 software. This study has been registered with PROSPERO.

**Results:**

A total of 35 studies involving 202,864 participants were analyzed. The overall prevalence of oral frailty among older adults was 34.0% (95% CI: 27.9–40.1%, *I*^2^ = 99.7%, *p* < 0.001). Subgroup analyses revealed statistically significant differences in the prevalence of oral frailty among different assessment tools and age groups (*p* < 0.05). Univariate meta-regression analysis indicated that the age was related to heterogeneity in the study (*p* < 0.05). Factors such as age, gender, physical frailty, pre-frailty, and unattached were identified as key risk factors for oral frailty in older adults (all *p* < 0.05).

**Conclusion:**

The incidence of oral frailty among older adults is notably high and influenced by a variety of factors. Healthcare professionals are encouraged to actively implement preventive and treatment measures addressing the controllable factors associated with oral frailty. Such proactive efforts are essential for early identification of high-risk individuals, which can help reduce the prevalence of oral frailty among older adults and enhance their quality of life.

**Systematic Review Registration:**

PROSPERO (CRD42023488653: https://www.crd.york.ac.uk/prospero/).

## 1 Introduction

With the intensification of global aging trends, frailty has become a major obstacle to achieving healthy aging (1). In this context, oral frailty has emerged as a distinct subtype of general frailty (2) and a novel concept within geriatric syndromes (3), attracting significant research attention. Defined as a progressive decline in oral health parameters including tooth count, oral function, and hygiene due to aging, oral frailty is accompanied by a reduced interest in maintaining oral health, diminished physiological and psychological reserves, and consequential impairments in eating functions (4). This overall decline precipitates further deterioration in both physical and mental health capacities (4).

Oral health is increasingly recognized as an essential component of overall health (5). As individuals age, oral cavity structures and functions inevitably deteriorate to varying degrees, which, combined with physical decline and pre-existing medical conditions, increases susceptibility to oral frailty (6). Oral frailty in older adults not only heightens the risk of physical frailty, disability, and even mortality (5, 7) but also serves as a risk factor for adverse health outcomes such as malnutrition, sarcopenia, falls, and cognitive impairments, significantly impacting their quality of life (5, 8–10). Therefore, understanding the prevalence and contributing factors of oral frailty among older adults is critical.

Current research reports a wide range of prevalence estimates for oral frailty among older adults, from 8.1 to 73.0% (11–13). This variability can be attributed to differences in assessment tools, participant age, and the country or region of the study. Although a recent systematic review on the prevalence of oral frailty in older adults has been conducted, it only included English-language studies, potentially missing relevant research in other languages, and primarily focused on prevalence without a comprehensive analysis of influencing factors (14). Identifying the factors influencing oral frailty in older adults is crucial for enhancing management strategies. However, existing research on these influencing factors remains inconsistent and unclear, necessitating a systematic and evidence-based review to clarify these associations.

To address these limitations, this study imposed no language restrictions and included multilingual literature (English, Chinese, and Japanese) to minimize the impact of language-related bias. In addition, the study conducted a systematic and comprehensive analysis of the influencing factors of oral frailty, offering novel insights into the complex interactions among its determinants. The findings aim to enhance healthcare providers’ understanding and awareness of oral frailty while providing evidence-based guidance for its early identification, prevention, and management in aging populations.

## 2 Materials and methods

This systematic review and meta-analysis was conducted in accordance with the MOOSE Reporting Guidelines for Meta-analyses of Observational Studies (15). The conduct and reporting also followed The PRISMA 2020 statement: an updated guideline for reporting systematic reviews (16). A detailed study protocol is available on the PROSPERO website under the registration number CRD42023488653.

### 2.1 Search strategy

A systematic search was conducted across multiple electronic databases, including PubMed, Embase, Web of Science, MEDLINE (via EBSCOhost), CINAHL, the Cochrane Library, Scopus, China Knowledge Resource Integrated Database (CNKI), Wanfang Database, Chinese Biomedical Database (CBM), and Weipu Database (VIP), from their inception through June 28, 2024. In addition, manual searches of reference lists from identified articles and related reviews were conducted to ensure the inclusion of all pertinent publications. Where data were not presented in a format amenable to meta-analysis, manuscript authors were contacted via email for clarification or additional information. The search strategy involved a combination of MeSH terms and free words, tailored to each database. The terms employed included “aged,” “old*,” “elder*,” “senior*,” “geriatric*,” and “oral frailty.” The precise search strategy for each English database is detailed in the appendix. This research did not require ethical committee approval, as it is entirely based on previously published studies. The details of the strategy are presented in Supplementary material S2.

### 2.2 Inclusion and exclusion criteria

The inclusion criteria for the systematic review were as follows: (a) Participants were older adults aged 60 years and above; (b) Studies used defined criteria and tools for assessing oral frailty, such as the Oral Frailty Index-6 (OFI-6), Oral Frailty Index-8 (OFI-8), or similar instruments; (c) Studies reported the prevalence or risk factors of oral frailty; (d) The study employed an observational design.

The exclusion criteria were as follows: (a) Studies that did not report relevant outcomes or had incomplete data; (b) Research classified as low-quality upon evaluation; (c) Types of publications such as reviews, conference abstracts, or patents; (d) Studies for which the full text could not be obtained; (e) In cases where multiple studies used the same cohort data at the same time point with identical criteria for oral frailty, only the study with the largest sample size and most comprehensive data was retained.

### 2.3 Study selection and data extraction

The process of study selection and data extraction was meticulously structured and conducted by two independent researchers. Initially, all identified literature was imported into NoteExpress for duplication checking. The researchers independently screened the titles, abstracts, and full texts of these studies, adhering strictly to the predetermined inclusion and exclusion criteria. Any discrepancies in the selection process were resolved through discussion or with the assistance of a third researcher.

For data extraction, the same two researchers independently extracted relevant data from the selected studies, ensuring accuracy through cross-verification. The extracted information included the first author’s name, publication year, study region, sample size, average age of participants, tools used for assessment, prevalence of oral frailty, and associated risk factors.

### 2.4 Quality evaluation of included studies

The quality assessment of the included studies was rigorously conducted by two independent reviewers, with any disagreements resolved through consultation with a third reviewer. For cohort studies, the Newcastle-Ottawa Scale (NOS) was used as the assessment tool. This scale evaluates studies across three domains: selection, comparability, and either outcome (for cohort studies) or exposure (for case–control studies). Points were awarded for each criterion, with up to one point for each item in the selection and exposure domains, and up to two points for comparability. The overall quality was categorized as follows: scores of 7–9 indicated high quality, 5–6 medium quality, and 0–4 low quality (17, 18). For cross-sectional studies, the Agency for Healthcare Research and Quality (AHRQ) criteria was applied. This 11-item checklist evaluates various aspects of the study, including source of information, inclusion and exclusion criteria, time period and continuity, blinding of personnel, quality assurance assessments, confounding and missing data, response rates, and patient completeness. Each item was scored as “1” for “Yes” and “0” for “No” or “Unclear.” The total score determined the study’s quality: scores of 8–11 indicated high quality, 4–7 medium quality, and 0–3 low quality (19).

### 2.5 Statistical analysis

Statistical analysis was performed using Stata 17.0 software. The prevalence of oral frailty was estimated using prevalence rates (%) along with their 95% confidence intervals (CI). For risk factors suitable for meta-analysis, odds ratios (ORs) and their 95% CI were used to quantify the association with oral frailty. Risk factors that were not amenable to meta-analysis were analyzed descriptively. Statistical heterogeneity among the studies was evaluated using the *I^2^* statistic. Considering the heterogeneity between studies, a random-effects model was applied for all analyses to allow for a more conservative inference of statistical significance (20). *p* < 0.05 was the threshold for statistical significance. In cases of substantial heterogeneity, subgroup analyses and meta-regression were utilized to explore the impact of study characteristics on the combined prevalence rates of oral frailty among older adults and to investigate the sources of heterogeneity. Sensitivity analysis was conducted to test the stability of the meta-analysis results for the combined prevalence rates of oral frailty. Publication bias was evaluated using funnel plots and Egger’s test; the absence of publication bias was inferred when *p* > 0.05, with evenly distributed funnel plot suggesting no bias. In the presence of publication bias, the trim and fill method was applied to adjust for the potential missing studies and to re-estimate the combined effect size.

## 3 Results

### 3.1 Study selection

**Figure 1:**
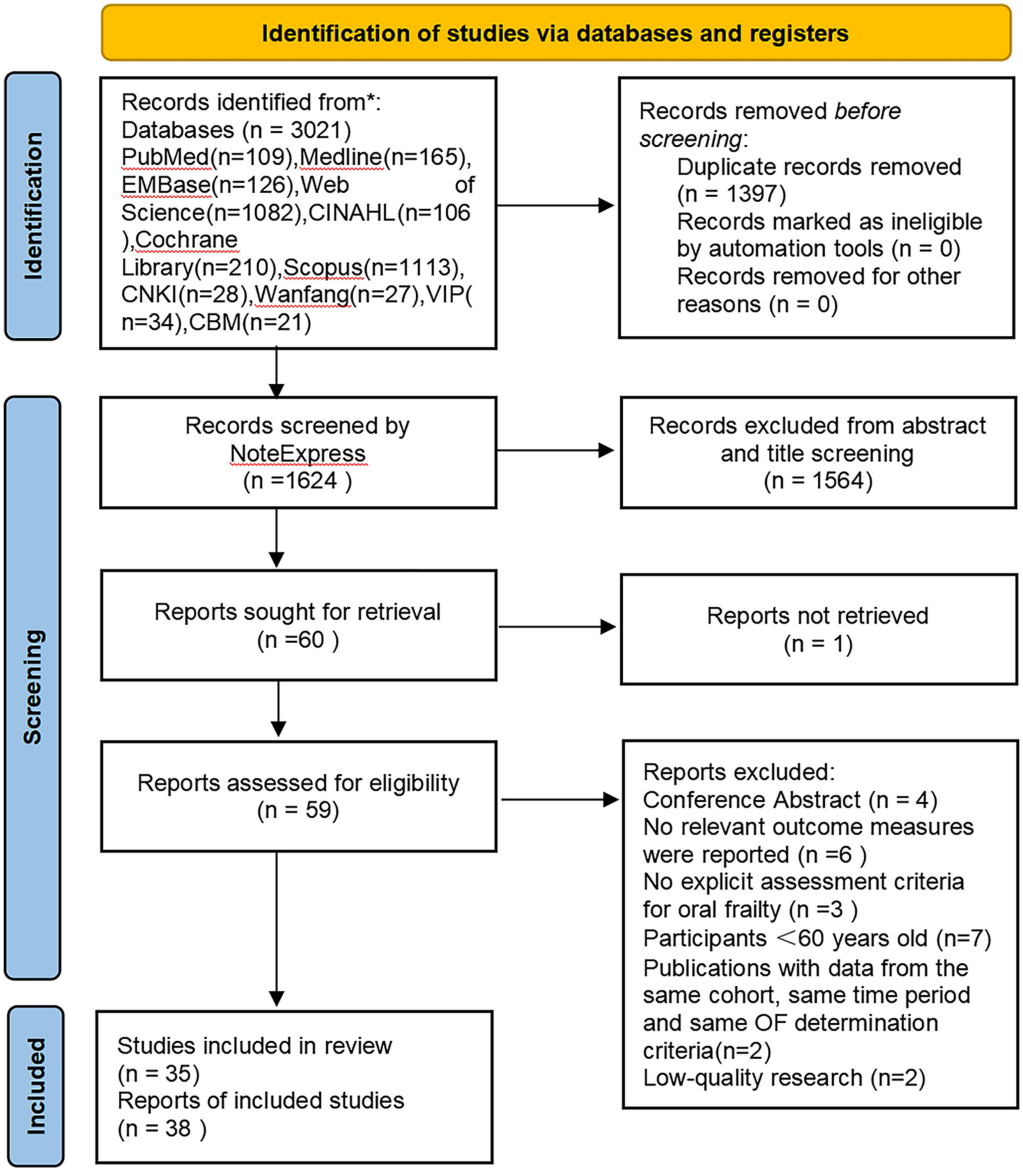
Flowchart of database search and study inclusion.

The initial database search yielded a total of 3,021 articles. Of these, 1,397 were identified as duplicates. Following the application of inclusion and exclusion criteria during title and abstract screening, 60 out of 1,624 articles were selected for full-text evaluation. This review process led to further exclusions: 4 studies were excluded due to their nature as conference abstracts; 6 were eliminated for not reporting relevant outcome measures; 3 were dismissed for lacking clear oral frailty assessment criteria; 7 were omitted due to the age of participants not meeting the inclusion criteria; 2 were excluded due to their low quality ratings; and 2 were excluded for utilizing data from the same cohort at the same time point with identical oral frailty assessment criteria. Furthermore, 1 study was excluded due to the unavailability of the full text. Consequently, 35 studies were ultimately included in the systematic review and meta-analysis. Figure 1 showed a selection process with the number of studies at each stage of the review process.

### 3.2 Characteristics and quality assessment of the included studies

The characteristics of the 35 studies included in our research are summarized in Table 1. These studies, published between 2018 and 2024, had a sample size ranging from 100 to 165,164 participants. Geographically, almost all of the studies were conducted in Asia: 24 from Japan (5–8, 10, 11, 21–38), 8 from China (12, 39–45), 1 from South Korea (13), and 1 from India (46). Only 1 study originated from Northern Europe—Finland (47). In terms of oral frailty assessment tools, 17 studies employed Oral Frailty Index-6 (OFI-6) (5–7, 10, 11, 24, 25, 30, 32–38, 45, 47), 10 studies utilized Oral Frailty Index-8 (OFI-8) (8, 12, 23, 31, 40–44, 46), and 3 studies adopted Oral Frailty 5-Item Checklist (OF-5) (22, 27, 29), representing the three most frequently used instruments. With the exception of 1 study (46), all studies reported the prevalence of oral frailty (5–8, 10–13, 21–45, 47), and 1 study presented prevalence data for oral frailty using four different assessment methods (41). Additionally, 12 studies identified risk factors associated with oral frailty (6, 7, 21, 25, 30, 32, 37, 41–44, 46). Regarding quality assessment, 31 cross-sectional studies (5–8, 11–13, 21, 22, 24–29, 31–34, 36–47) were evaluated using the AHRQ scoring system, resulting in 18 high-quality (6, 7, 12, 21, 28, 29, 33, 34, 36, 37, 39–46) and 13 medium-quality (5, 8, 11, 13, 22, 24–27, 31, 32, 38, 47) publications (Supplementary material S3). The remaining 4 studies (10, 23, 30, 35) were assessed using the NOS scoring system and were all categorized as high-quality research (Supplementary material S3).

**Table 1:** Characteristics of included studies.

Author/year	Country	Mean age	Sample size	Prevalence of oral frailty (%)	Oral frailty assessment	Risk factors assessed	Quality level
Yamamoto et al. (21)	Japan	≥65	165,164	51.7	Model for oral frailty prediction	female, age, separated/divorced, educational attainment, equivalent income, frequency of meeting friends, social cohesion, civic participation, ruralagricultural	H
Iwasaki et al. (22)	Japan	74.7 ± 5.5	1,206	36.7	OF-5	NR	M
Watanabe et al. (23)	Japan	73.6 ± 6.0	11,374	62.6	OFI-8	NR	H
Arai et al. (24)	Japan	≥75	2,190	44.4	OFI-6	NR	M
Nakagawa et al. (25)	Japan	≥75	2,727	44.3	OFI-6	Simplified nutritional appetite questionnaire, dietary variety score, age, female, frailty, No. medications taken a day	M
Fei et al. (39)	China	≥60	307	17.3	TN+OFI-8+ODK	NR	H
Kang et al. (13)	Korea	78.0 ± 7.4	100	73.0	KAGD screening questionnaire and diagnostic criteria	NR	M
Kawamura et al. (26)	Japan	77.2 ± 5.7	111	37.8	Seven items to evaluate oral frailty	NR	M
Song et al. (40)	China	71.89 ± 7.58	409	41.3	OFI-8	NR	H
Miyahara et al. (27)	Japan	77.6 ± 6.8	248	46.0	OF-5	NR	M
Yin et al. (41)	China	≥60	310	69.0	OFI-8	Passive smoking	H
Yin et al. (41)	China	≥60	310	27.4	OFI-8+TN	Being widowed/unmarried	H
Yin et al. (41)	China	≥60	310	51.9	OFI-8+ODK	Pre-frailty, physical frailty	H
Yin et al. (41)	China	≥60	310	21.0	OFI-8+TN+ODK	80 years old and above, being widowed/unmarried	H
Julkunen et al. (47)	Finland	NR	303	53.0	OF-checklist	NR	M
Kimura et al. (28)	Japan	74.3 ± 6.1	208	8.2	OFI-6	NR	H
Kumar et al. (46)	India	66.72 ± 6.86	310	NR	OFI-8	Age	H
Kusunoki et al. (8)	Japan	77.70 ± 6.60	251	38.6	OFI-8	NR	M
Tanaka et al. (29)	Japan	73.10 ± 5.60	2031	39.3	OF-5	NR	H
Nishimoto et al. (30)	Japan	72.20 ± 5.10	1,234	NR	OFI-6	Severe periodontitis	H
Nagatani et al. (10)	Japan	72.40 ± 5.20	1,410	16.9	OFI-6	NR	H
Kamide et al. (31)	Japan	76.00 ± 5.70	237	54.9	OFI-8	NR	M
Tang et al. (42)	China	72.70 ± 6.30	1,298	44.7	OFI-8	Female, age, chronic diseases, depressive symptoms, low level of social support, meat-based diet, salty taste	H
Wang et al. (43)	China	≥60	223	59.2	OFI-8	Age, personal monthly income, smoking, oral health-related self-efficacy	H
Tu et al. (44)	China	72.71 ± 8.00	204	33.8	OFI-8	Age, gender, education level, polypharmacy, physical frailty, number of dentures, dry mouth, subjective masticatory difficulty, and oral health	H
Kuo and Lee (12)	China/Taiwan	79.70 ± 7.20	308	60.4	OFI-8	NR	H
Baba et al. (11)	Japan	74.20 ± 6.10	210	8.1	OFI-6	NR	M
Lin et al. (45)	China/Taiwan	≥65	1,100	20.7	OFI-6	NR	H
Yamamoto et al. (32)	Japan	77.9 ± 5.4	843	25.5	OFI-6	Age ≥ 85, number of teeth present < 20, difficulty eating tough foods, choking	M
Hoshino et al. (33)	Japan	75.90 ± 6.30	481	21.2	OFI-6	NR	H
Komatsu et al. (6)	Japan	72.80 ± 5.50	380	14	OFI-6	Physical frailty risk, gait speed	H
Iwasaki et al. (34)	Japan	77.10 ± 4.70	1,082	21	OFI-6	NR	H
Tanaka et al. (35)	Japan	≥65	1,301	24.4	OFI-6	NR	H
Nishimoto et al. (36)	Japan	76.30 ± 5.10	940	8.4	OFI-6	NR	H
Hironaka et al. (7)	Japan	73.30 ± 6.60	682	9.5	OFI-6	Age, mini nutritional assessment short form score, stroke, number of medications used, social frailty, pre-frailty	H
Ohara et al. (37)	Japan	79.10 ± 4.50	722	19.3	OFI-6	Eating alone	H
Kugimiya et al. (38)	Japan	76.30 ± 6.50	679	22.5	OFI-6	NR	M
Tanaka et al. (5)	Japan	73.00 ± 5.50	2011	16	OFI-6	NR	M

OFI-6, Oral Frailty Index-6; OFI-8, Oral Frailty Index-8; OF-5, Oral Frailty 5-Item Checklist; OF checklist, Oral Frailty checklist; TN, the Number of Natural Teeth; ODK, Oral Diadochokinesis; NR, not report; H, high quality; M, medium quality.

### 3.3 Prevalence of oral frailty

**Figure 2:**
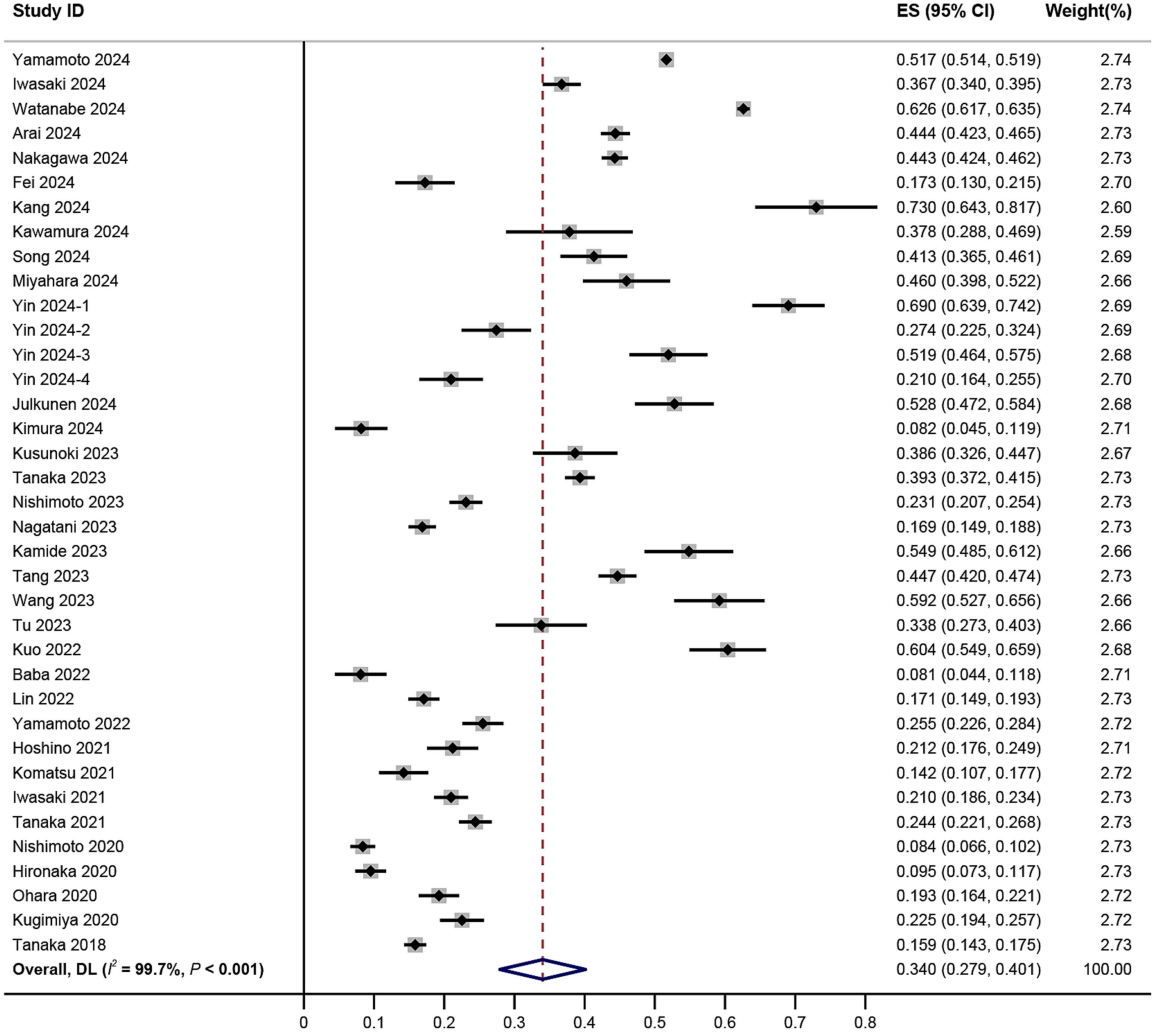
Forest plot of prevalence of oral frailty among older adults.

**Figure 3:**
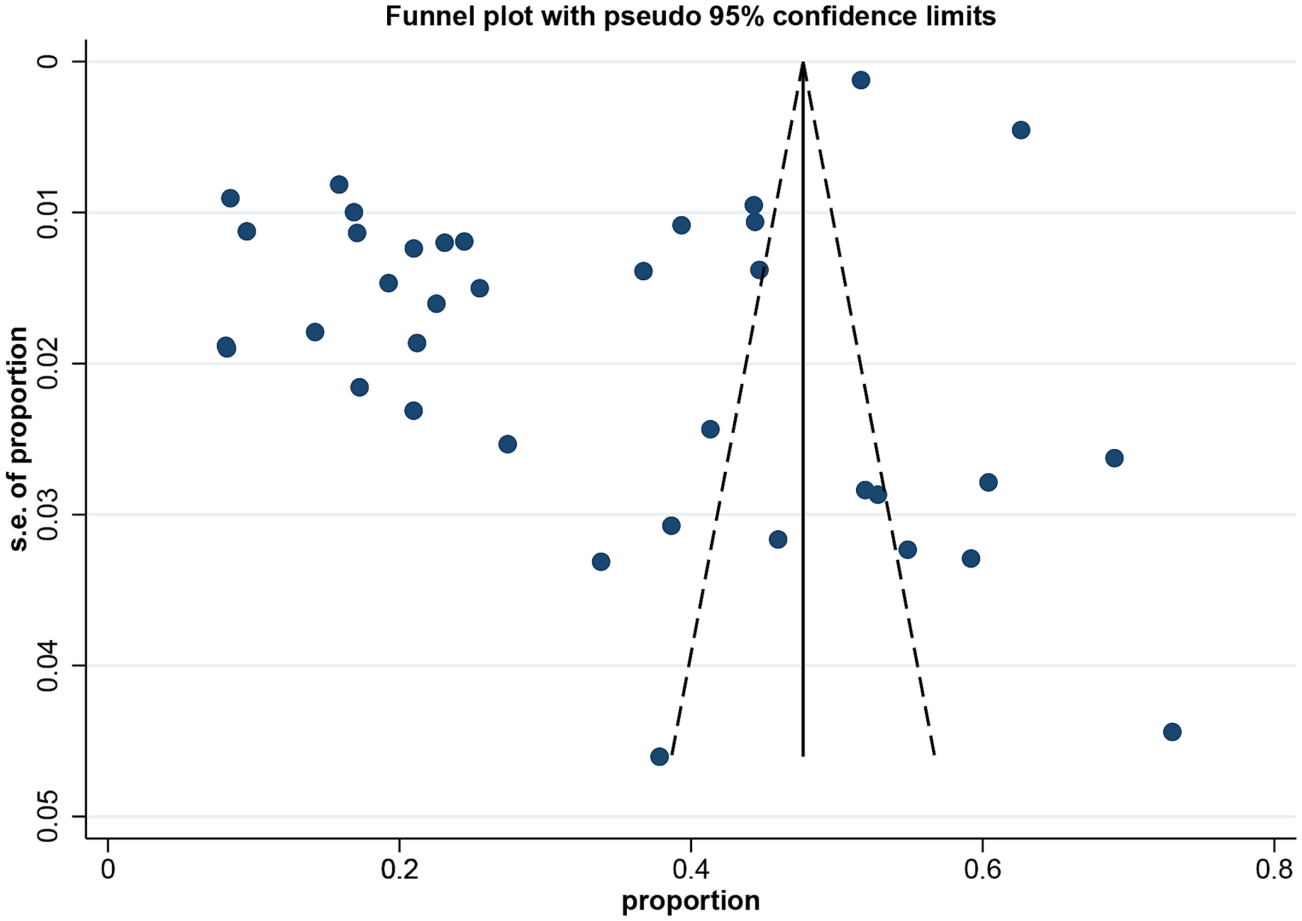
Funnel plot for assessing publication biases.

In the 34 studies (5–8, 10–13, 21–45, 47) eligible for our meta-analysis, the prevalence of oral frailty varied widely, ranging from 8.1 to 73.0%. Given the substantial heterogeneity observed (*I*^2^ = 99.7%, *p* < 0.001), a random-effects model was employed to estimate the pooled prevalence. Our analysis indicated that the combined prevalence of oral frailty was 34.0% (95% CI, 27.9–40.1%, *I*^2^ = 99.7%, *p* < 0.001) (Figure 2). The visual inspection of the funnel plot, as depicted in Figure 3, revealed asymmetry, and the findings from Egger’s test further indicated the potential presence of publication bias (*t* = −4.39, *p* < 0.001).

### 3.4 Publication bias testing

To assess potential publication bias, we first conducted a visual inspection using a funnel plot (Figure 3). The plot showed asymmetry, suggesting the presence of publication bias. Further Egger’s test revealed a significant small study effect (*t* = −4.39, *p* < 0.001), which further supported the finding of potential publication bias. To address this bias, we applied the trim and fill method for correction. The results indicated no studies needed to be trimmed, and the combined effect size before and after the correction remained consistent, with no significant impact on the overall results (Supplementary Figure S1).

### 3.5 Sensitivity analyses

Sensitivity analysis confirmed the robustness of our findings, as sequential exclusion of individual studies did not significantly alter the pooled prevalence (Supplementary Figure S2). This consistency highlights the reliability of the meta-analysis results, even in the presence of high heterogeneity. However, future studies should address the methodological and contextual differences contributing to the observed heterogeneity.

### 3.6 Subgroup analyses and meta-regression

To explore the sources of heterogeneity, we performed subgroup analyses based on assessment tools, gender, age, geographic region, and population source (Table 2). Additionally, meta-regression was employed to quantitatively evaluate the effects of these covariates on between-study variance (Table 3).

**Table 2:** Subgroup analysis of the prevalence of oral frailty in older adults.

Subgroups	Number of included reports	Oral frailty	Heterogeneity		Difference between groups	
		Prevalence (95% CI) (%)	*I* ^2^	*p* value	Qbet	*p* value
**Scales for OF**					102.00	<0.001
OFI-6	17	20.3 (14.5, 26.0)	99.0%	<0.001		
OFI-8	9	51.7 (43.7, 59.6)	97.6%	<0.001		
OF-5	3	39.7 (36.0, 43.3)	73.6%	0.023		
OFI-8+TN+ODK	2	19.0 (15.4, 22.6)	27.1%	0.241		
**Gender**					0.54	0.463
Male	26	32.6 (24.9, 40.3)	99.5%	<0.001		
Female	26	36.4 (29.9, 42.9)	99.4%	<0.001		
**Age**					12.60	0.002
60–69	9	35.2 (27.2, 43.3)	96.2%	<0.001		
70–79	9	44.0 (36.1, 51.9)	97.1%	<0.001		
≥80	9	60.9 (49.2, 72.6)	94.8%	<0.001		
**Country**					2.74	0.098
Japan	24	28.9 (21.2, 36.6)	99.8%	<0.001		
China	11	40.2 (29.2, 51.2)	98.7%	<0.001		
**Source**					1.21	0.547
Community	27	32.1 (23.8, 40.4)	99.7%	<0.001		
Healthcare or long-term care facility	5	40.1 (28.4, 51.7)	95.7%	<0.001		
Rural	4	35.4 (17.4, 53.4)	99.1%	<0.001		

**Table 3:** Meta-regression results on the prevalence of oral frailty in older adults.

Covariates	Coefficient	95% CI	*p* value	𝜏^2^
Assessment tool	0.063	(−0.023, 0.150)	0.147	0.02978
Gender	−0.038	(−0.141, 0.064)	0.455	0.03272
Age	0.126	(0.039, 0.213)	0.006	0.02849
Country	−0.113	(−0.238, 0.012)	0.074	0.02791
Source	0.029	(−0.065, 0.123)	0.533	0.03397

Subgroup analyses revealed different pooled prevalence rates of oral frailty depending on the assessment tools used: 51.7% for OFI-8, 20.3% for OFI-6, 39.7% for OF-5, and 19.0% for a combination of OFI-8, NT, and ODK. Furthermore, the estimated pooled prevalence of oral frailty was higher among older females (36.4, 95% CI: 29.9–42.9%) compared to older males (32.6, 95% CI: 24.9–40.3%). With respect to age, the prevalence estimates for oral frailty were 35.2% for individuals aged 60–69, 44.0% for those aged 70–79, and 60.9% for those aged 80 and above. Geographically, the pooled prevalence of oral frailty was higher in the Chinese older population (40.2, 95% CI: 29.2–51.2%) compared to their Japanese counterparts (28.9, 95% CI: 21.2–36.6%). Lastly, the prevalence of oral frailty among older adults in healthcare or long-term care facilities (40.1, 95% CI: 28.4–51.7%) was higher compared to those in community-based settings (32.1, 95% CI: 23.8–40.4%) and rural populations (35.4, 95% CI: 17.4–53.4%). These results suggest that assessment tools and age may partially explain the observed heterogeneity.

Meta-regression further evaluated the effects of these factors. Among the variables analyzed, only age was statistically significant, reducing between-study variance from 0.0354 to 0.02849, accounting for 25.47% of the heterogeneity (Table 3). This finding aligns with subgroup analysis results, confirming that older age groups are associated with higher prevalence of oral frailty. However, a substantial portion of the heterogeneity remained unexplained. The assessment tool, gender, country of publication, and source of population did not demonstrate significant explanatory power for heterogeneity, as evidenced by their non-significant regression results.

### 3.7 Risk factors

In our comprehensive analysis, a total of 35 risk factors were evaluated across 12 included studies. Among these, six risk factors—age, gender, physical frailty, pre-frailty, polypharmacy and unattached (referring to being separated/divorced, widowed/unmarried, or eating alone)—were consistently identified in multiple studies (at least two). With the exception of polypharmacy, all these factors were found to be significantly associated with the prevalence of oral frailty in older adults (*p* < 0.05), as detailed in Table 4.

**Table 4:** Pooled risk factors of oral frailty in older adults.

Risk factors	Number of included studies	Heterogeneity		Meta-analysis	
		*I* ^2^	*p* value	OR (95% CI)	*p* value
Age	4	37.1%	0.190	1.09 (1.08, 1.11)	<0.001
Female	3	31.7%	0.321	1.53 (1.27, 1.85)	<0.001
Physical frailty	4	71.2%	0.015	2.47 (1.31, 4.65)	0.005
Pre-frailty	2	0.0%	0.978	1.75 (1.07, 2.87)	0.025
Polypharmacy	3	59.5%	0.085	1.09 (0.98, 1.22)	0.119
Unattached	4	78.2%	0.003	1.75 (1.06, 2.90)	0.029

In addition to the six consistently reported risk factors, our review identified 29 additional risk factors from single studies. These factors span multiple domains, including: (1) Oral health factors: severe periodontitis, oral health-related self-efficacy, number of teeth (<20), dry mouth, subjective chewing difficulties, and choking; (2) Lifestyle and dietary habits: poor sleep quality, dietary variety score, preference for meat-based diets, smoking, and passive smoking; (3) Nutritional status: scores on the mini nutritional assessment-short form and the simplified nutritional appetite questionnaire; (4) Social and psychological factors: depressive symptoms, low social support, social frailty, and civic participation; (5) Economic and educational factors: personal monthly income and educational level; (6) Health status and physical function: chronic illnesses, gait speed, and stroke. These factors were reported in individual studies and cannot be combined in a meta-analysis due to a lack of overlap across studies. However, they highlight the multifactorial nature of oral frailty, encompassing physical, psychosocial, and lifestyle domains.

## 4 Discussion

In this systematic review and meta-analysis, which synthesized data from 34 studies involving 202,864 individuals aged 60 and above, we determined that the estimated pooled prevalence of oral frailty stands at 34.0% (95% CI: 27.9–40.1%, *I*^2^ = 99.7%, *p* < 0.001). This rate significantly exceeds those reported in prior research (14), a discrepancy likely attributable to our inclusion of a more extensive array of literature, including newer studies that encompass a broader geographical and demographic scope. The considerable prevalence highlights oral frailty as a critical public health issue due to its associated with adverse outcomes such as malnutrition, diminished quality of life, and elevated mortality risk (5, 9, 48). Moreover, the findings illuminate the complexity of worsening oral health in older adults and corroborate existing research that portrays oral frailty as an integral component of the broader spectrum of age-related frailty (2). Additionally, despite the funnel plot showing asymmetry and Egger’s test indicating the presence of publication bias, the application of the Trim and Fill method did not change the combined effect size. Sensitivity analysis also confirmed the robustness of the results, suggesting that even in the presence of potential publication bias, its impact on the conclusions of this study is limited.

The results of subgroup analysis and meta-regression analysis indicate that the significant heterogeneity observed among the studies (*I*^2^ = 99.7%) can primarily be attributed to three factors. Firstly, the operational definitions and diagnostic criteria for oral frailty varied significantly across studies, resulting in notable discrepancies in the reported prevalence rates. Our findings suggest that the Oral Frailty Index-8 (OFI-8) generally reported higher prevalence rates compared to the Oral Frailty Index-6 (OFI-6) and other methodologies. This may be due to the OFI-8’s comprehensive assessment approach, which evaluates functional oral health, oral health-related behaviors, and social engagement (35). Despite their frequent use, OFI-6 and OFI-8 differ in sensitivity and specificity, potentially capturing varying degrees of oral health issues. The lack of sufficient validation for these tools underscores the urgent need for a standardized oral frailty assessment tool, especially for cross-cultural and cross-regional applications. Future research should prioritize the standardization and extensive validation of these tools to improve the comparability and reliability of study results.

Secondly, subgroup analyses revealed that the pooled prevalence of oral frailty among older adults in China (40.2, 95% CI: 29.2–51.2%) was higher than in Japan (28.9, 95% CI: 21.2–36.6%), with several factors contributing to this disparity. Healthcare system differences are notable, as Japan offers comprehensive oral healthcare programs, including routine check-ups and geriatric oral health promotion (49), while China has limited oral healthcare coverage, especially in rural areas with inadequate access to dental and preventive services (50). Cultural factors also play a role, as Japanese older adults exhibit higher oral health literacy and adherence to preventive practices, such as regular brushing and dental visits, supported by public education (49), whereas the limited oral health knowledge among Chinese older adults hinders behavioral changes, indirectly affecting their oral health status (51, 52). Additionally, methodological differences contribute, as studies in China predominantly use the Oral Frailty Index-8 (OFI-8), which incorporates broader functional and behavioral criteria, leading to higher prevalence rates, while Japanese studies typically use the Oral Frailty Index-6 (OFI-6), focusing on more specific physiological indicators. This geographical variability highlights the importance of tailoring public health strategies to the specific needs of different populations.

Finally, the meta-regression results highlighted that age could explain up to 25.47% of the heterogeneity observed in our study. Notably, the prevalence of oral frailty increases significantly with age, with pooled prevalence rates rising from 35.2% in individuals aged 60–69 to 60.9% in those aged 80 and above. This progression underscores the impact of aging on oral health deterioration and aligns with gerontological research which suggests that physiological and social changes associated with aging can markedly affect oral health outcomes (53). Consequently, it is crucial for relevant stakeholders to develop comprehensive and stratified screening programs targeting oral frailty in older populations across diverse age groups, thereby enabling the timely implementation of preventive and interventional measures. Despite our analysis of these factors, the heterogeneity remains high. This suggests that other potential contributing factors, such as differences in study design, sample selection, and data collection methods, may also be responsible for the observed heterogeneity. Further research may need to focus on standardizing these aspects to mitigate the impact of heterogeneity.

Diverging from previous study, our current systematic and meta-analysis undertook a comprehensive and nuanced analysis of a broad spectrum of risk factors, thereby enriching our understanding of the prevalence and underlying mechanisms of oral frailty in older adults. The meta-analysis results on risk factors indicate that age, gender, polypharmacy, and physical frailty are significant factors contributing to oral frailty in older adults. The correlation between these factors and oral frailty may reflect the complex interplay between systemic health and oral conditions.

The prevalence of oral frailty among older adults increases with advancing age. Aging is well-established as a significant risk factor for various forms of frailty. Physiological changes associated with aging, such as reduced saliva production and tooth loss, compromise the mouth’s defense mechanisms against infections, thereby impairing oral health (54). Reduced bone density, particularly in the alveolar bones supporting the teeth, often exacerbates this deterioration, increasing the likelihood of tooth mobility and eventual loss (55). Systemic health issues that accompany aging might directly impact the maintenance of oral health through the side effects of medications used in treatment. Moreover, cognitive decline commonly observed in older adults can impair their ability to perform effective daily oral hygiene, increasing the risk of periodontal disease and cavities (56). To address the complexities of aging and its impact on oral health, healthcare providers, particularly geriatricians and dentists, must incorporate regular oral health assessments into routine care for older adults. Early intervention and tailored care plans can help mitigate these age-related changes and their adverse effects on oral health, thereby enhancing the overall quality of life for older adults. Furthermore, continuous education should be provided to caregivers and patients to underscore the importance of maintaining oral hygiene even amidst physical and cognitive declines.

Gender significantly influences the prevalence and manifestations of oral frailty among older adults, with women showing a higher prevalence compared to their male counterparts. This discrepancy is largely attributed to hormonal changes that affect oral health. For instance, estrogen deficiency associated with menopause is linked to decreased bone mineral density, including that of the alveolar bones supporting the teeth, thereby increasing the risk of tooth loss and periodontal disease (57). Additionally, women are more susceptible to autoimmune diseases such as Sjögren’s syndrome, which further impairs salivary function and oral health (58). Understanding these unique challenges faced by women in maintaining oral health can inform more targeted interventions, thus alleviating the burden of oral frailty and improving overall well-being in this population.

Physical frailty in older adults, characterized by declines in muscle strength, endurance, and overall activity levels (5), significantly impacts oral health by limiting the ability to maintain proper oral hygiene and manage nutritional needs, thereby creating a cyclical relationship with oral frailty. The inherent decline in physical capabilities associated with frailty reduces the effectiveness of daily oral hygiene tasks. Reduced flexibility and muscle weakness make effective brushing and flossing challenging, increasing the risk of oral diseases. This deterioration in oral health can lead to pain and discomfort, which exacerbates difficulties in eating, thereby affecting nutritional intake and overall health (59, 60). Physical frailty often coexists with declining nutritional status, and malnutrition weakens the immune system, reduces saliva production, and increases susceptibility to oral infections, contributing to oral frailty (61). Moreover, physical frailty is often linked to other age-related conditions, such as osteoporosis and arthritis, which can indirectly impact oral health. Within the frailty spectrum, pre-frailty represents a critical early stage, where individuals begin to exhibit physical limitations but have not yet reached full frailty. Recognizing and addressing pre-frailty is crucial as it presents an opportunity for intervention before significant impacts on oral health occur (62). Given these intricate connections, managing physical frailty in older adults must include strategies to support oral health, such as adapting oral hygiene tools for easier use, incorporating regular dental assessments into elderly care, and educating caregivers on the importance of oral health in the overall management of frailty.

Being unattached, which includes individuals who are separated, divorced, widowed, or unmarried, significantly impacts oral health in older adults, with the primary reasons possibly being the lack of social support and increased feelings of isolation that often accompany these statuses. Social support is instrumental in maintaining health behaviors, notably in regular dental care and effective daily oral hygiene practices (63). In contrast, unattached older adults often experience reduced psychosocial stimulation and may suffer from mental health challenges such as loneliness or depression, which can lead to neglect of personal health care, including oral hygiene (64). The lack of a partner not only diminishes emotional support but also practical assistance in managing healthcare routines, making this population more susceptible to oral frailty (65). Research indicates that older adults without companionship are less likely to seek regular dental care and are more prone to dietary habits that negatively impact oral health (66). Therefore, recognizing the unique needs of unattached older adults is essential for developing targeted interventions that address both their oral health and broader psychosocial needs.

Our analysis also identified an additional 29 factors influencing oral frailty, each reported in individual studies, covering a broad spectrum from oral health conditions to lifestyle habits and socioeconomic status. Severe periodontitis and dentures directly impair an individual’s chewing ability, thereby affecting nutritional intake (67). Smoking exacerbates periodontal disease and disrupts the oral microbiome, while insufficient sleep leads to systemic inflammation and impaired healing responses, further deteriorating oral health (68, 69). Moreover, dietary habits play a pivotal role. A preference for a meat-centric diet can lead to nutritional imbalances that affect oral health (70). Social and psychological factors, including symptoms of depression and low levels of social support, indirectly impact oral health by influencing personal care routines and dietary choices, potentially leading to neglected oral hygiene and poor dietary habits, thus exacerbating oral frailty (71, 72). Additionally, economically disadvantaged groups commonly face reduced access to healthcare and lower health literacy, hindering effective management and prevention of oral diseases, thereby increasing the risk of oral frailty (73). These findings underscore the need for a comprehensive approach to managing oral health in older adults, which should incorporate not only direct medical interventions but also lifestyle modifications, psychological support, and socioeconomic measures. Such an all-encompassing care strategy is essential for mitigating the various risks associated with oral frailty and enhancing the overall health status of the aging population.

## 5 Strengths and limitations

Overall, the strengths of our study include a comprehensive search across 11 databases to minimize the risk of missing relevant studies. In addition to providing an estimate of the pooled prevalence of oral frailty, our research is the first to conduct a comprehensive analysis of its influencing factors. Moreover, we explored potential sources of heterogeneity through sensitivity analysis, subgroup analysis, and meta-regression analysis, which enhance the robustness of our findings.

However, this study also has certain limitations. Firstly, there is considerable heterogeneity among the included studies. Heterogeneity is often inevitable in meta-analyses of observational studies and does not necessarily invalidate the results, but we can only attempt to explore potential sources of heterogeneity as far as possible. Secondly, an in-depth analysis of some risk factors for oral frailty was not feasible due to the limited number of studies covering these factors. Although we conducted preliminary descriptive analyses, the limited number of included studies restricted the robustness of the conclusions. Future research should focus on increasing sample sizes, incorporating data from diverse cultural and regional contexts, and standardizing assessment tools to enhance the consistency and comparability of findings. Furthermore, subgroup analyses of these factors require more extensive data to ensure sufficient statistical power and reliability. Thirdly, as most studies in this research are concentrated in Asia, particularly Japan and China, the findings may have limited global applicability. While these regions are representative of oral frailty in older adults, cultural, socioeconomic, and healthcare policy differences may influence prevalence and risk factors. Future studies should include data from other regions to assess the broader applicability of these findings across different populations and better understand regional variations. Finally, despite a comprehensive database search, potential related studies, such as unpublished and gray literature, may still have been missed.

## 6 Conclusion

In conclusion, this systematic review identified that the pooled prevalence of oral frailty among older adults is 34.0%. Such a high prevalence rate indicates a need to raise public awareness about oral frailty in older adults. Risk factors for oral frailty include age, gender, physical frailty, pre-frailty, being unattached, and 29 factors involving oral health, lifestyle and dietary habits, nutritional status, social and psychological factors, economic and educational factors, and health status and physical function. These findings suggest that oral frailty among older adults is characterized by high prevalence and complexity, and future prevention and management should focus on these associated risk factors.

To effectively tackle oral frailty, interventions should include regular oral health assessments, health education, psychosocial support, and multidisciplinary collaboration. Regular oral health check-ups facilitate early detection and intervention, while health education can raise awareness and encourage self-care and healthy eating habits. A multidisciplinary approach, integrating geriatrics, dentistry, nutrition, and other fields, is essential for comprehensive management and has proven successful in countries like the United States and the United Kingdom. For older adults who are isolated or lack social support, psychological support and facilitated social activities can improve mental well-being and promote healthier lifestyles. Furthermore, comparing intervention models from other countries can provide valuable insights for developing public health strategies tailored to China and other East Asian nations. By incorporating a combination of these interventions and drawing on international experiences, future public health strategies are more likely to reduce the burden of oral frailty and improve the overall health of older adults. Our findings provide evidence-based support for advancing oral frailty research, offer practical guidance for healthcare professionals, and inform public health policies aimed at mitigating the impact of oral frailty and enhancing the health and quality of life of older adults.

## Data Availability

The original contributions presented in the study are included in the article/Supplementary material, further inquiries can be directed to the corresponding author.

## Supplementary material

SUPPLEMENTARY Figure S1 Trim and fill plot for publication bias adjustment.

SUPPLEMENTARY Figure S2 Sensitivity analysis of pooled effect estimates.

## References

[1] Wernly B Bruno RR Beil M Flaatten H Kelm M Sigal S . Frailty's influence on 30-day mortality in old critically ill ICU patients: a bayesian analysis evaluating the clinical frailty scale. Ann Intensive Care. (2023) 13:126. doi: 10.1186/s13613-023-01223-9 38091131 PMC10719192

[2] Ayoob A Neelamana S Janakiram C. Impact of oral frailty on general frailty in geriatric population. J Indian Assoc Public Health Dent. (2022) 20:9–15. doi: 10.4103/jiaphd.jiaphd_91_21

[3] PayneM MorleyJE. Editorial: dysphagia, dementia and frailty. J Nutr Health Aging. (2018) 22:562–5. doi: 10.1007/s12603-018-1033-5, PMID: 29717753

[4] Japan Dental Association. Manual for oral frailty. (2020). Available at: https://www.jda.or.jp/oral_frail/2020/pdf/2020-manual-all.pdf (Accessed July 17, 2024).

[5] Tanaka T Takahashi K Hirano H Kikutani T Watanabe Y Ohara Y . Oral frailty as a risk factor for physical frailty and mortality in community-dwelling elderly. J Gerontol A Biol Sci Med Sci. (2018) 73:1661–7. doi: 10.1093/gerona/glx225, PMID: 29161342

[6] Komatsu R Nagai K Hasegawa Y Okuda K Okinaka Y Wada Y . Association between physical frailty subdomains and Oral frailty in community-dwelling older adults. Int J Environ Res Public Health. (2021) 18:2931. doi: 10.3390/ijerph18062931, PMID: 33809322 PMC8001836

[7] Hironaka S Kugimiya Y Watanabe Y Motokawa K Hirano H Kawai H . Association between oral, social, and physical frailty in community-dwelling older adults. Arch Gerontol Geriatr. (2020) 89:104105. doi: 10.1016/j.archger.2020.104105, PMID: 32480111

[8] Kusunoki H Ekawa K Kato N Yamasaki K Motone M Shinmura K . Association between oral frailty and cystatin C-related indices-a questionnaire (OFI-8) study in general internal medicine practice. PLoS One. (2023) 18:e0283803. doi: 10.1371/journal.pone.0283803, PMID: 37093792 PMC10124892

[9] IwasakiM MotokawaK WatanabeY ShirobeM InagakiH EdahiroA . Association between Oral frailty and nutritional status among community-dwelling older adults: the Takashimadaira study. J Nutr Health Aging. (2020) 24:1003–10. doi: 10.1007/s12603-020-1433-1, PMID: 33155629

[10] Nagatani M Tanaka T Son BK Kawamura J Tagomori J Hirano H . Oral frailty as a risk factor for mild cognitive impairment in community-dwelling older adults: Kashiwa study. Exp Gerontol. (2023) 172:112075. doi: 10.1016/j.exger.2022.112075, PMID: 36581224

[11] Baba H Watanabe Y Miura K Ozaki K Matsushita T Kondoh M . Oral frailty and carriage of oral Candida in community-dwelling older adults (check-up to discover health with energy for senior residents in Iwamizawa; CHEER Iwamizawa). Gerodontology. (2022) 39:49–58. doi: 10.1111/ger.12621, PMID: 35098575

[12] Kuo YW Lee JD. Association between Oral frailty and physical frailty among rural middle-old community-dwelling people with cognitive decline in Taiwan: a cross-sectional study. Int J Environ Res Public Health. (2022) 19:2884. doi: 10.3390/ijerph19052884, PMID: 35270577 PMC8909940

[13] Kang JH Park SC Jung HI Jung SJ Park HJ Kim SM . Validation of the Korean academy of geriatric dentistry screening questionnaire and oral frailty diagnostic criteria in community-dwelling older adults. Epidemiol Health. (2024) 46:e2024008. doi: 10.4178/epih.e2024008, PMID: 38186249 PMC11099569

[14] Li T Shen Y Leng Y Zeng Y Li L Yang Z . The prevalence of oral frailty among older adults: a systematic review and meta-analysis. Eur Geriatr Med. (2024) 15:645–55. doi: 10.1007/s41999-023-00930-7, PMID: 38528284

[15] Brooke BS Schwartz TA Pawlik TM. MOOSE reporting guidelines for Meta-analyses of observational studies. JAMA Surg. (2021) 156:787–8. doi: 10.1001/jamasurg.2021.0522 33825847

[16] Page MJ McKenzie JE Bossuyt PM Boutron I Hoffmann TC Mulrow CD . The PRISMA 2020 statement: an updated guideline for reporting systematic reviews. BMJ. (2021) 372:n71. doi: 10.1136/bmj.n71, PMID: 33782057 PMC8005924

[17] Ottawa: Ottawa Hospital Research Institute. The Newcastle-Ottawa scale (NOS) for assessing the quality of nonrandomized studies in meta-analyses. (2024). Available at: http://www.ohri.ca/programs/clinical_epidemiology/oxford.htm (Accessed July 17, 2024).

[18] Yuan C Li W Liu J Li J. Frailty and transplant-free survival of patients with liver cirrhosis: a meta-analysis. PLoS One. (2024) 19:e0302836. doi: 10.1371/journal.pone.0302836, PMID: 38722913 PMC11081249

[19] Song WX Wu WW Zhao YY Xu HL Chen GC Jin SY . Evidence from a meta-analysis and systematic review reveals the global prevalence of mild cognitive impairment. Front Aging Neurosci. (2023) 15:1227112. doi: 10.3389/fnagi.2023.1227112, PMID: 37965493 PMC10641463

[20] Schmidt FL Oh IS Hayes TL. Fixed-versus random-effects models in meta-analysis: model properties and an empirical comparison of differences in results. Br J Math Stat Psychol. (2009) 62:97–128. doi: 10.1348/000711007X255327 18001516

[21] Yamamoto T Mochida Y Irie K Altanbagana NU Fuchida S Aida J . Regional inequalities in Oral frailty and social capital. JDR Clin Trans Res. (2024) 9:368–77. doi: 10.1177/23800844241238648 38654451

[22] Iwasaki M Shirobe M Motokawa K Tanaka T Ikebe K Ueda T . Prevalence of Oral frailty and its association with dietary variety, social engagement, and physical frailty: results from the Oral frailty 5-item checklist. Geriatr Gerontol Int. (2024) 24:371–7. doi: 10.1111/ggi.14846, PMID: 38390632

[23] Watanabe D Yoshida T Watanabe Y Yokoyama K Yamada Y Kikutani T . Oral frailty is associated with mortality independently of physical and psychological frailty among older adults. Exp Gerontol. (2024) 191:112446. doi: 10.1016/j.exger.2024.112446, PMID: 38679352

[24] Arai E Watanabe Y Nakagawa S Ohara Y Iwasaki M Hirano H . Association of oral frailty with medical expenditure in older Japanese adults: the study of late-stage older adults in Tottori (START Tottori). Gerodontology. (2024). doi: 10.1111/ger.12771, PMID: 38887126

[25] Nakagawa S Miura K Arai E Taira K Watanabe Y Shirobe M . Oral frailty, appetite and dietary variety in late-stage older adults: a cross-sectional study (the STudy of lAte-stage oldeR adulTs in Tottori; START Tottori). Geriatr Gerontol Int. (2024) 24:626–33. doi: 10.1111/ggi.14892, PMID: 38714504

[26] Kawamura K Maeda K Miyahara S Shimizu A Ishida Y Ueshima J . Association between oral frailty and sarcopenia among frailty clinic outpatients: a cross-sectional study. Nutrition. (2024) 124:112438. doi: 10.1016/j.nut.2024.112438, PMID: 38657417

[27] Miyahara S Maeda K Kawamura K Matsui Y Onaka M Satake S . Concordance in oral frailty five-item checklist and oral hypofunction: examining their respective characteristics. Arch Gerontol Geriatr. (2024) 118:105305. doi: 10.1016/j.archger.2023.105305, PMID: 38056104

[28] Kimura C Miura K Watanabe Y Baba H Ozaki K Hasebe A . Association between oral frailty andPrevotellapercentage in the oral microbiota of community‐dwelling older adults who participated in theCHEERIwamizawa project, Japan. J Oral Rehabil. (2024) 51:1721–9. doi: 10.1111/joor.13767, PMID: 38850071

[29] Tanaka T Hirano H Ikebe K Ueda T Iwasaki M Shirobe M . Oral frailty five-item checklist to predict adverse health outcomes in community-dwelling older adults: a Kashiwa cohort study. Geriatr Gerontol Int. (2023) 23:651–9. doi: 10.1111/ggi.14634, PMID: 37661091 PMC11503571

[30] Nishimoto M Tanaka T Hirano H Watanabe Y Ohara Y Shirobe M . Severe periodontitis increases the risk of Oral frailty: a six-year follow-up study from Kashiwa cohort study. Geriatrics (Basel). (2023) 8:25. doi: 10.3390/geriatrics8010025, PMID: 36826367 PMC9956982

[31] Kamide N Ando M Murakami T Sawada T Hata W Sakamoto M. The association of oral frailty with fall risk in community-dwelling older adults: a cross-sectional, observational study. Eur Geriatr Med. (2024) 15:279–83. doi: 10.1007/s41999-023-00863-1, PMID: 37697213

[32] Yamamoto T Tanaka T Hirano H Mochida Y Iijima K. Model to predict Oral frailty based on a questionnaire: a cross-sectional study. Int J Environ Res Public Health. (2022) 19:13244. doi: 10.3390/ijerph192013244, PMID: 36293822 PMC9603718

[33] HoshinoD HiranoH EdahiroA MotokawaK ShirobeM WatanabeY . Association between Oral frailty and dietary variety among community-dwelling older persons: a cross-sectional study. J Nutr Health Aging. (2021) 25:361–8. doi: 10.1007/s12603-020-1538-633575729

[34] Iwasaki M Watanabe Y Motokawa K Shirobe M Inagaki H Motohashi Y . Oral frailty and gait performance in community-dwelling older adults: findings from the Takashimadaira study. J Prosthodont Res. (2021) 65:467–73. doi: 10.2186/jpr.JPR_D_20_00129 33612666

[35] Tanaka T Hirano H Ohara Y Nishimoto M Iijima K. Oral frailty Index-8 in the risk assessment of new-onset oral frailty and functional disability among community-dwelling older adults. Arch Gerontol Geriatr. (2021) 94:104340. doi: 10.1016/j.archger.2021.104340, PMID: 33529863

[36] Nishimoto M Tanaka T Takahashi K Unyaporn S Fujisaki-Sueda-Sakai M Yoshizawa Y . Oral frailty is associated with food satisfaction in community-dwelling older adults. Nihon Ronen Igakkai Zasshi. (2020) 57:273–81. doi: 10.3143/geriatrics.57.273 32893209

[37] Ohara Y Motokawa K Watanabe Y Shirobe M Inagaki H Motohashi Y . Association of eating alone with oral frailty among community-dwelling older adults in Japan. Arch Gerontol Geriatr. (2020) 87:104014. doi: 10.1016/j.archger.2020.104014, PMID: 32000053

[38] Kugimiya Y Watanabe Y Ueda T Motokawa K Shirobe M Igarashi K . Rate of oral frailty and oral hypofunction in rural community-dwelling older Japanese individuals. Gerodontology. (2020) 37:342–52. doi: 10.1111/ger.12468, PMID: 32141117

[39] Fei Y Niu S Xi X Tang W Zhao Y Zhang G . Physical frailty intensifies the positive association of oral frailty with poor global cognitive function and executive function among older adults especially for females: a cross-sectional study. BMC Geriatr. (2024) 24:468. doi: 10.1186/s12877-024-05056-4, PMID: 38811863 PMC11134949

[40] Song H Wei Y Wang Y Zhang J. The mediating effect of nutrition on oral frailty and fall risk in community-dwelling elderly people. BMC Geriatr. (2024) 24:273. doi: 10.1186/s12877-024-04889-3, PMID: 38504156 PMC10953286

[41] Yin Y Zhao Y Fei Y Liu Y Ji Y Shan E . Epidemiology and risk factors of oral frailty among older people: an observational study from China. BMC Oral Health. (2024) 24:368. doi: 10.1186/s12903-024-04149-1, PMID: 38515048 PMC10958975

[42] Tang J Tang X Zeng L Chen H Yang X Zhou Q . Prevalence and influencing factors of oral frailty in the elderly of rural areas in Guizhou Province. Chin J Prev Control Chronic Dis. (2023) 5:327–31. doi: 10.16386/j.cjpccd.issn.1004-6194.2023.05.002

[43] Wang L Ju M Wang T Wang F Pu X Jia X . Oral frailty risk and its influencing factors in community-dwelling elderly population. J Nurs Sci. (2023) 18:112–6.

[44] Tu H Zhang S Fang Y He G. Current situation and influencing factors of oral frailty in the community elderly. Chin J Nurs. (2023) 11:1351–6.

[45] Lin YC Huang SS Yen CW Kabasawa Y Lee CH Huang HL. Physical frailty and Oral frailty associated with late-life depression in community-dwelling older adults. J Pers Med. (2022) 12:459. doi: 10.3390/jpm12030459, PMID: 35330459 PMC8954826

[46] Kumar G Dash P Jena S. Assessment of prosthetic status and Oral frailty among the geriatric population residing in old age homes of Bhubaneswar City-a cross sectional study. J Health Sci Med Res. (2023):2023941. doi: 10.31584/jhsmr.2023941

[47] Julkunen L Saarela R Roitto HM Kautiainen H Pitkälä K Mäntylä P . Oral frailty among dentate and edentate older adults in long-term care. BMC Geriatr. (2024) 24:48. doi: 10.1186/s12877-023-04605-7, PMID: 38212720 PMC10782602

[48] Dibello V Lobbezoo F Lozupone M Sardone R Ballini A Berardino G . Oral frailty indicators to target major adverse health-related outcomes in older age: a systematic review. Geroscience. (2023) 45:663–706. doi: 10.1007/s11357-022-00663-8, PMID: 36242694 PMC9886742

[49] Okamoto E. Japan's dental care facing population aging: how universal coverage responds to the changing needs of the elderly. Int J Environ Res Public Health. (2021) 18:9359. doi: 10.3390/ijerph18179359, PMID: 34501951 PMC8430920

[50] Qi X Qu X Wu B. Urban-rural disparities in dental services utilization among adults in China's megacities. Front Oral Health. (2021) 2:673296. doi: 10.3389/froh.2021.673296, PMID: 35048016 PMC8757718

[51] An R Jiang G Wu Z Liu M Sohaib M Chen W. Perceptions and experience of rural older people in oral health management in China: a qualitative study. BMC Oral Health. (2024) 24:644. doi: 10.1186/s12903-024-04401-8, PMID: 38822319 PMC11143558

[52] Cao C Liao S Cao W Guo Y Hong Z Ren B . Differences in the association of oral health knowledge, attitudes, and practices with frailty among community-dwelling older people in China. BMC Oral Health. (2023) 23:782. doi: 10.1186/s12903-023-03477-y, PMID: 37875820 PMC10594714

[53] Wang M Deng X Chen H Diao Y Liu C Gao J . Frailty mediated the association between tooth loss and mortality in the oldest old individuals: a cohort study. Front Public Health. (2024) 11:1285226. doi: 10.3389/fpubh.2023.1285226, PMID: 38328540 PMC10848322

[54] Fornari CB Bergonci D Stein CB Agostini BA Rigo L. Prevalence of xerostomia and its association with systemic diseases and medications in the elderly: a cross-sectional study. Sao Paulo Med J. (2021) 139:380–7. doi: 10.1590/1516-3180.2020.0616.R3.1902021, PMID: 34190871 PMC9615591

[55] Zhu L Tang Z Hu R Gu M Yang Y. Ageing and inflammation: what happens in periodontium? Bioengineering (Basel). (2023) 10:1274. doi: 10.3390/bioengineering10111274, PMID: 38002398 PMC10669535

[56] Nakamura T Zou K Shibuya Y Michikawa M. Oral dysfunctions and cognitive impairment/dementia. J Neurosci Res. (2021) 99:518–28. doi: 10.1002/jnr.24745, PMID: 33164225

[57] Adam M Wooton J. Menopause and oral health. Br Dent J. (2022) 233:170. doi: 10.1038/s41415-022-4568-0 35962073

[58] American Dental Association. Sjögren disease. (2024). Available at: https://www.ada.org/resources/ada-library/oral-health-topics/sjogren-disease (Accessed July 17, 2024).

[59] Liu S Guo Y Hu Z Zhou F Li S Xu H. Association of oral status with frailty among older adults in nursing homes: a cross-sectional study. BMC Oral Health. (2023) 23:368. doi: 10.1186/s12903-023-03009-8, PMID: 37287021 PMC10249201

[60] Palacios C Joshipura KJ. Nutrition and oral health: a two-way relationship In: Handbook of clinical nutrition and aging. New York: Springer New York (2015). 81–98.

[61] Sheetal A Hiremath VK Patil AG Sajjansetty S Kumar SR. Malnutrition and its oral outcome-a review. J Clin Diagn Res. (2013) 7:178–80. doi: 10.7860/JCDR/2012/5104.2702, PMID: 23449967 PMC3576783

[62] Sacha J Sacha M Soboń J Borysiuk Z Feusette P. Is it time to begin a public campaign concerning frailty and pre-frailty? A review article. Front Physiol. (2017) 8:484. doi: 10.3389/fphys.2017.00484, PMID: 28744225 PMC5504234

[63] Tsakos G Sabbah W Chandola T Newton T Kawachi I Aida J . Social relationships and oral health among adults aged 60 years or older. Psychosom Med. (2013) 75:178–86. doi: 10.1097/PSY.0b013e31827d221b, PMID: 23324876

[64] Perissinotto CM Stijacic Cenzer I Covinsky KE. Loneliness in older persons: a predictor of functional decline and death. Arch Intern Med. (2012) 172:1078–83. doi: 10.1001/archinternmed.2012.1993 PMID: 22710744, PMID: 22710744 PMC4383762

[65] Inoue Y Zaitsu T Oshiro A Ishimaru M Taira K Takahashi H . Association of marital status and access to dental care among the Japanese population: a cross-sectional study. BMC Oral Health. (2022) 22:278. doi: 10.1186/s12903-022-02311-1, PMID: 35799162 PMC9264690

[66] Aida J Kondo K Kondo N Watt RG Sheiham A Tsakos G. Income inequality, social capital and self-rated health and dental status in older Japanese. Soc Sci Med. (2011) 73:1561–8. doi: 10.1016/j.socscimed.2011.09.005, PMID: 21982631

[67] O'Keeffe M Kelly M O'Herlihy E O'Toole PW Kearney PM Timmons S . Potentially modifiable determinants of malnutrition in older adults: a systematic review. Clin Nutr. (2019) 38:2477–98. doi: 10.1016/j.clnu.2018.12.007, PMID: 30685297

[68] Huang C Shi G. Smoking and microbiome in oral, airway, gut and some systemic diseases. J Transl Med. (2019) 17:225. doi: 10.1186/s12967-019-1971-7, PMID: 31307469 PMC6632217

[69] Carra MC Schmitt A Thomas F Danchin N Pannier B Bouchard P. Sleep disorders and oral health: a cross-sectional study. Clin Oral Investig. (2017) 21:975–83. doi: 10.1007/s00784-016-1851-y, PMID: 27178314

[70] Kotronia E Brown H Papacosta AO Lennon LT Weyant RJ Whincup PH . Poor oral health and the association with diet quality and intake in older people in two studies in the UK and USA. Br J Nutr. (2021) 126:118–30. doi: 10.1017/S0007114521000180, PMID: 33468264 PMC8187263

[71] Goh V Hassan FW Baharin B Rosli TI. Impact of psychological states on periodontitis severity and oral health-related quality of life. J Oral Sci. (2022) 64:1–5. doi: 10.2334/josnusd.21-0267, PMID: 34690248

[72] Berglund E Westerling R Lytsy P. Social and health-related factors associated with refraining from seeking dental care: a cross-sectional population study. Community Dent Oral Epidemiol. (2017) 45:258–65. doi: 10.1111/cdoe.12284, PMID: 28169442

[73] Corovic S Janicijevic K Radovanovic S Vukomanovic IS Mihaljevic O Djordjevic J . Socioeconomic inequalities in the use of dental health care among the adult population in Serbia. Front Public Health. (2023) 11:1244663. doi: 10.3389/fpubh.2023.1244663, PMID: 37790713 PMC10545090

