# Supplementary figures and images for: Systematic review and meta-analysis on the prevalence and risk factors of oral frailty among older adults
Source: Front Med (Lausanne). 2025 Jan 22;12:1512927. doi: 10.3389/fmed.2025.1512927 (PMC11794213; doi:10.3389/fmed.2025.1512927)

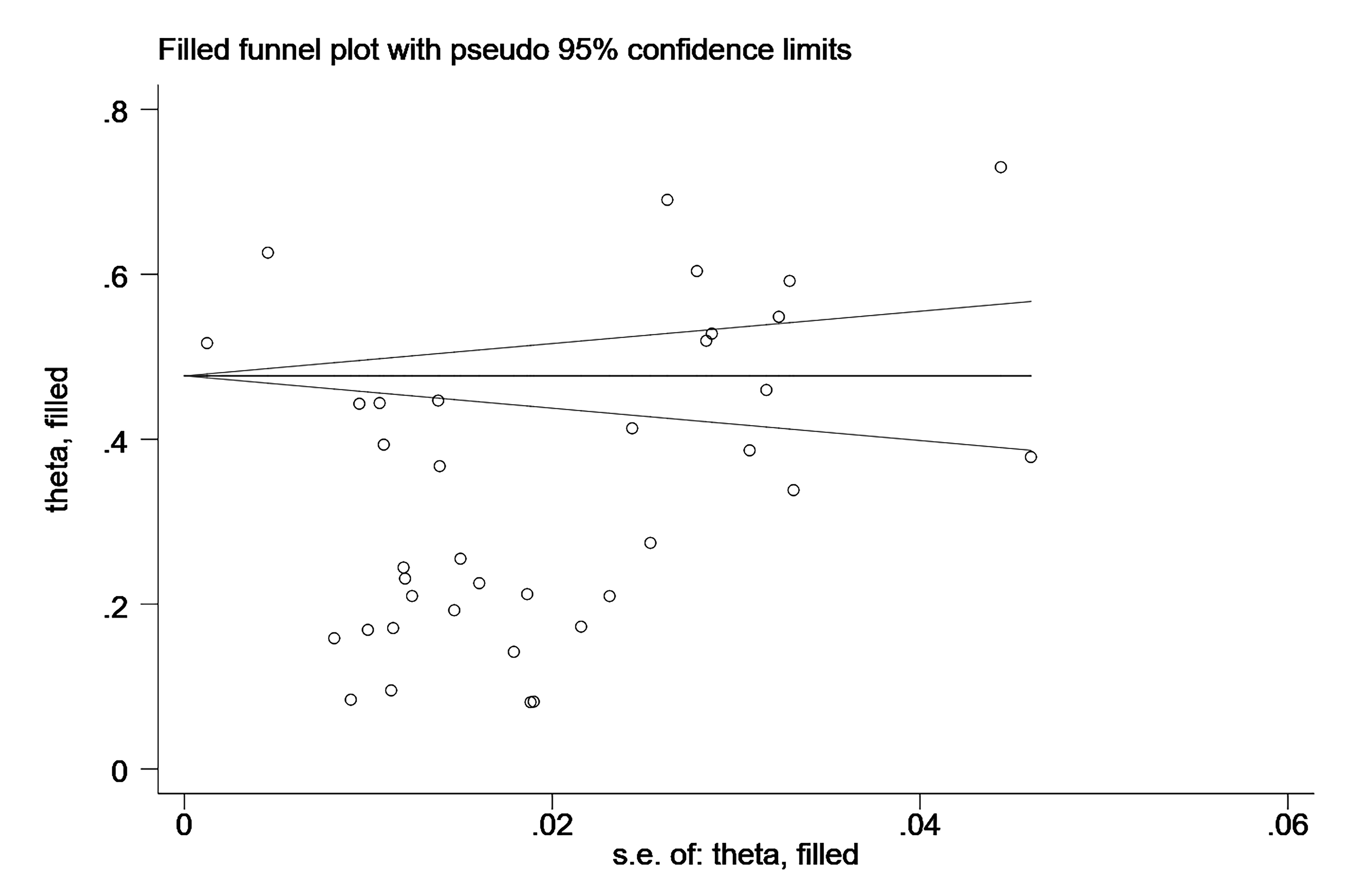

Supplement: SUPPLEMENTARY Figure S1 — Trim and fill plot for publication bias adjustment. [file Image_1.tif]

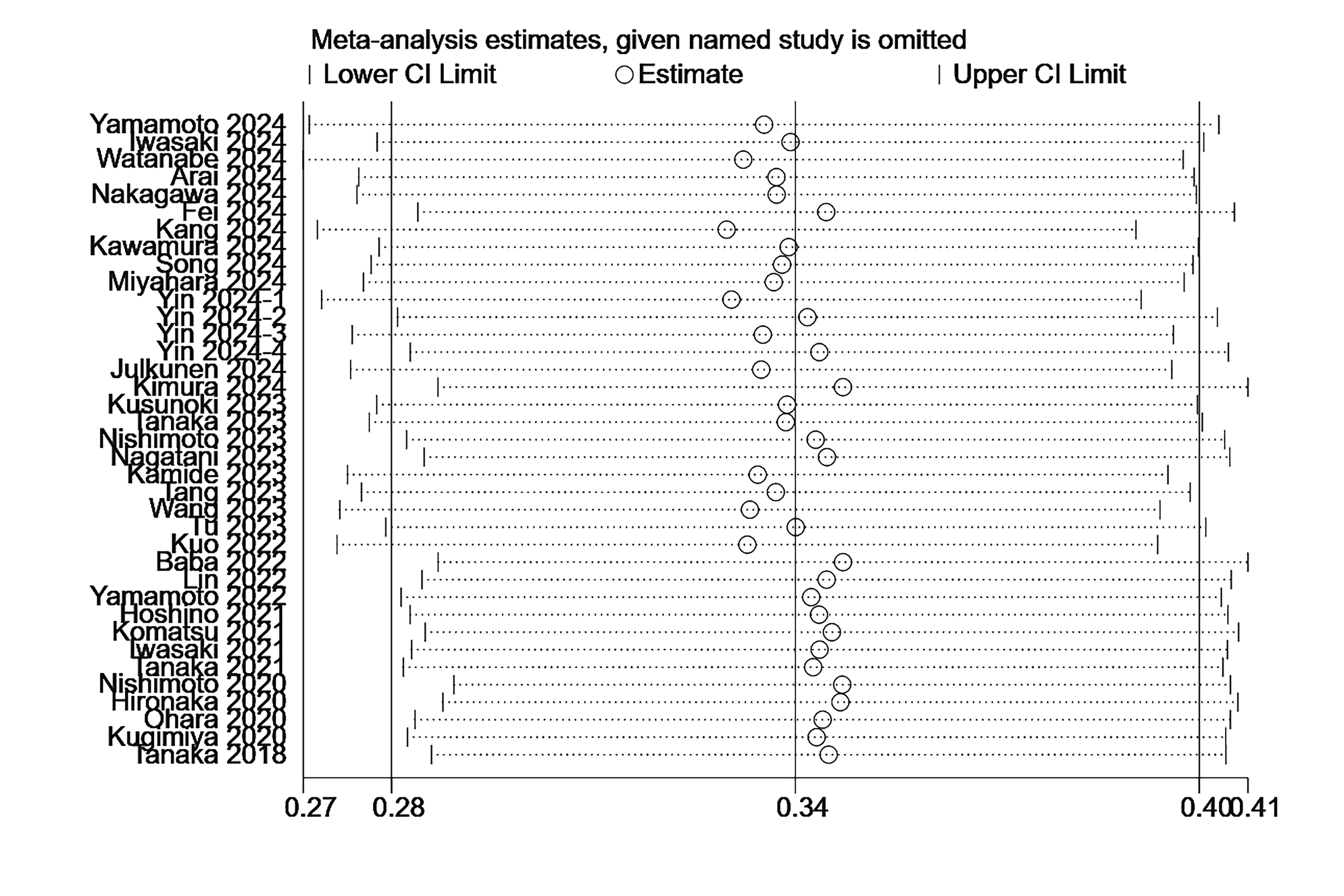

Supplement: SUPPLEMENTARY Figure S2 — Sensitivity analysis of pooled effect estimates. [file Image_2.tif]
