# Supplementary material for: Systematic review and meta-analysis on the prevalence and risk factors of oral frailty among older adults
Source: Front Med (Lausanne). 2025 Jan 22;12:1512927. doi: 10.3389/fmed.2025.1512927 (PMC11794213; doi:10.3389/fmed.2025.1512927)
Supplement: Supplementary file 3 [file Data_Sheet_1.PDF]

Search strategy

PubMed

#1 ((((aged[MeSH Terms]) OR (old*[Title/Abstract])) OR (elder*[Title/Abstract])) OR (senior*[Title/Abstract])) OR (geriatric*[Title/Abstract])

4,770,857

#2 oral frailty[Title/Abstract] 135

#3 #1 AND #2 (oral frailty[Title/Abstract]) AND (((((aged[MeSH Terms]) OR (old[Title/Abstract])) OR (elder*[Title/Abstract])) OR (senior*[Title/Abstract])) OR (geriatric*[Title/Abstract])) 109

A total of 109 articles

Embase

#1 'aged'/exp OR old*:ti,ab,kw OR elder*:ti,ab,kw OR senior*:ti,ab,kw OR geriatric*:ti,ab,kw 6,171,904

#2 'oral frailty':ti,ab,kw 139

#3 #1 And #2 126

A total of 126 articles

Web of Science

#1 aged (主题) or old* (主题) or elder* (主题) or senior* (主题) or geriatric* (主题) 5,951,809

#2 oral frailty (主题) 1,231

#3 #1 AND #2 1,082

A total of 1,082 articles

MEDLINE (via EBSCOhost)

#1 MM aged OR AB old* OR AB elder* OR AB senior* OR AB geriatric* 763,643

#2 TI old* OR TI elder* OR TI senior* OR TI geriatric* 220,381

#3 #1 OR #2 820,633

#4 AB oral frailty OR TI oral frailty 195

#5 #3 AND #4 165

A total of 165 articles

CINAHL

#1 MM aged OR TI old* OR TI elder* OR TI senior* OR TI geriatric* 142,530

#2 AB old* OR AB elder* OR AB senior* OR AB geriatric* 270,217

#3 #1 OR #2 332,595

#4 TI oral frailty OR AB oral frailty 124

#5 #3 AND #4 106

A total of 106 articles

Cochrane Library

#1 MeSH descriptor: [Aged] in all MeSH products 275454

#2 (old*):ti,ab,kw OR (elder*):ti,ab,kw OR (senior*):ti,ab,kw OR

(geriatric*):ti,ab,kw 208429

#3 #1 OR #2 439733

#4 (oral frailty):ti,ab,kw 291

#5 #3 AND #4 210

A total of 210 articles

Scopus

#1 (TITLE-ABS-KEY (aged) OR TITLE-ABS-KEY (old*) OR TITLE-ABS-KEY (elder*) OR TITLE-ABS-KEY (senior*) OR TITLE-ABS-KEY (geriatric*)) 8666860

#2 TITLE-ABS-KEY (oral AND frailty) 1303

#3 #1 AND #2（((TITLE-ABS-KEY (aged) OR TITLE-ABS-KEY (old*) OR TITLE-ABS-KEY (elder*) OR TITLE-ABS-KEY (senior*) OR TITLE-ABS-KEY (geriatric*))) AND (TITLE-ABS-KEY (oral AND frailty))） 1113

A total of 1,113 articles

China Knowledge Resource Integrated Database (CNKI)

(SU='老年人'+'老人'+'老年'+'老年患者'+'老年病人'+'老年人群') and (SU='口腔衰弱') 28

A total of 28 articles

Wanfang Database

(主题:("老年人" or "老人" or "老年" or "老年患者" or "老年病人" or "

老年人群") and 主题:("口腔衰弱")) 27

A total of 27 articles

Chinese Biomedical Database (CBM)

#1 "老年人"[不加权:扩展] 168

#2 "老人"[摘要:智能] OR "老年"[摘要:智能] OR "老年患者"[摘要:智能] OR "老年病人"[摘要:智能] OR "老年人群"[摘要:智能] 433041

#3 "老人"[中文标题:智能] OR "老年"[中文标题:智能] OR "老年患者"[中文标题:智能] OR "老年病人"[中文标题:智能] OR "老年人群"[中文标题:智能] 374364

#4 (#3) OR (#2) OR (#1) 497558

#5 "口腔衰弱"[摘要:智能] OR "口腔衰弱"[中文标题:智能] 23

#6 (#5) AND (#4)21

A total of 21 articles

Weipu Database (VIP)

M=(老年人 OR 老人 OR 老年 OR 老年患者 OR 老年病人 OR 老年人群) AND M=(口腔衰弱) 34

A total of 34 articles
