# Supplementary material for: Systematic review and meta-analysis on the prevalence and risk factors of oral frailty among older adults
Source: Front Med (Lausanne). 2025 Jan 22;12:1512927. doi: 10.3389/fmed.2025.1512927 (PMC11794213; doi:10.3389/fmed.2025.1512927)
Supplement: Supplementary file 4 [file Data_Sheet_2.PDF]

Table A

Methodological quality appraisal results based on the AHRQ tool for each study.

| Stady | Item 1 | Item 2 | Item 3 | Item 4 | Item 5 | Item 6 | Item 7 | Item 8 | Item 9 | Item 10 | Item 11 | Total score | Quality |
|---|---|---|---|---|---|---|---|---|---|---|---|---|---|
| Yamamoto 2024 | Y | Y | Y | Y | U | Y | Y | Y | Y | Y | U | 9 | H |
| Iwasaki 2024 | Y | Y | Y | U | U | N | Y | Y | Y | Y | U | 7 | M |
| Arai 2024 | Y | Y | Y | Y | U | U | N | Y | Y | Y | U | 7 | M |
| Nakagawa 2024 | Y | N | Y | Y | U | U | Y | Y | Y | Y | U | 7 | M |
| Fei 2024 | Y | Y | Y | Y | U | Y | Y | Y | Y | Y | U | 9 | H |
| Kang 2024 | Y | Y | N | U | U | Y | Y | U | Y | Y | U | 6 | M |
| Kawamura 2024 | Y | Y | Y | U | U | Y | Y | Y | Y | U | U | 7 | M |
| Song 2024 | Y | Y | Y | U | U | Y | Y | Y | Y | Y | U | 8 | H |
| Miyahara 2024 | Y | Y | Y | U | U | Y | Y | Y | U | Y | U | 7 | M |
| Yin 2024 | Y | Y | Y | Y | U | Y | Y | Y | Y | Y | U | 9 | H |
| Julkunen 2024 | Y | Y | U | U | U | Y | Y | Y | U | Y | U | 6 | M |
| Kimura 2024 | Y | Y | Y | U | U | Y | Y | Y | Y | Y | U | 8 | H |
| Kumar 2023 | Y | Y | Y | Y | Y | Y | Y | Y | U | U | U | 8 | H |
| Kusunoki 2023 | Y | N | Y | Y | Y | Y | N | N | U | U | U | 6 | M |
| Tanaka 2023 | Y | Y | Y | U | Y | Y | Y | Y | Y | Y | Y | 10 | H |
| Kamide 2023 | Y | Y | Y | Y | U | U | Y | Y | U | U | U | 6 | M |
| Tang 2023 | Y | Y | Y | Y | Y | Y | Y | Y | U | U | U | 8 | H |
| Wang 2023 | Y | Y | Y | Y | Y | Y | Y | Y | U | Y | U | 9 | H |
| Tu 2023 | Y | Y | Y | Y | Y | Y | Y | Y | Y | Y | U | 10 | H |
| Kuo 2022 | Y | Y | Y | Y | Y | Y | Y | Y | Y | Y | U | 10 | H |
| Baba 2022 | Y | N | N | N | Y | Y | Y | Y | Y | Y | U | 7 | M |

| | | | | | | | | | | | | | |
|---|---|---|---|---|---|---|---|---|---|---|---|---|---|
| Lin 2022 | Y | Y | Y | Y | Y | Y | Y | Y | U | Y | U | 9 | H |
| Yamamoto 2022 | Y | Y | Y | U | N | U | Y | Y | U | U | U | 5 | M |
| Hoshino 2021 | Y | Y | Y | Y | Y | Y | Y | Y | Y | Y | U | 10 | H |
| Komatsu 2021 | Y | Y | Y | N | Y | Y | Y | Y | Y | U | U | 8 | H |
| Iwasaki 2021 | Y | Y | Y | U | Y | Y | Y | Y | Y | Y | U | 9 | H |
| Nishimoto 2020 | Y | Y | Y | U | Y | Y | Y | Y | Y | U | U | 8 | H |
| Hironaka 2020 | Y | Y | Y | U | Y | Y | Y | Y | Y | Y | U | 9 | H |
| Ohara 2020 | Y | U | Y | U | Y | Y | Y | Y | Y | Y | U | 8 | H |
| Kugimiya 2020 | Y | Y | Y | U | Y | Y | Y | Y | U | U | U | 7 | M |
| Tanaka 2018 | Y | Y | Y | U | Y | Y | Y | Y | U | U | U | 7 | M |

*Note:* Y, yes; N, no; U, unclear; H, high quality; M, medium quality.

Item 1: Define the source of information (survey, record review).

Item 2: List inclusion and exclusion criteria for exposed and unexposed subjects (cases and controls) or refer to previous publications.

Item 3: Indicate time period used for identifying patients.

Item 4: Indicate whether or not subjects were consecutive if not population-based.

Item 5: Indicate if evaluators of subjective components of study were masked to other aspects of the status of the participants.

Item 6: Describe any assessments undertaken for quality assurance purposes (e.g., test/retest of primary outcome measurements).

Item 7: Explain any patient exclusions from analysis.

Item 8: Describe how confounding was assessed and/or controlled.

Item 9: If applicable, explain how missing data were handled in the analysis.

Item 10: Summarize patient response rates and completeness of data collection.

Item 11: Clarify what follow-up, if any, was expected and the percentage of patients for which incomplete data or follow-up was obtained.

Table B

Methodological quality appraisal results based on the NOS tool for each study.

| **Stady** | **Selection** | | | | **Comparability** | **Outcome** | | | **Total** | **Quality** |
|---|---|---|---|---|---|---|---|---|---|---|
| | **Item 1** | **Item 2** | **Item 3** | **Item 4** | **Item 5** | **Item 6** | **Item 7** | **Item 8** | | |
| Watanabe 2024 | ✯ | ✯ | ✯ | ✯ | ✯✯ | ✯ | ✯ | ✯ | 9 | H |
| Nishimoto 2023 | ✯ | ✯ | ✯ | ✯ | ✯✯ | ✯ | ✯ | ✯ | 9 | H |
| Nagatani 2023 | ✯ | ✯ | ✯ | ✯ | ✯✯ | ✯ | ✯ | ✯ | 9 | H |
| Tanaka 2021 | ✯ | ✯ | ✯ | ✯ | ✯✯ | ✯ | ✯ | | 8 | H |

*Note:* H, high quality; M, medium quality.

Item 1: Representativeness of the exposed cohort.

Item 2: Selection of the non exposed cohort.

Item 3: Ascertainment of exposure.

Item 4: Demonstration that outcome of interest was not present at start of study.

Item 5: Comparability of cohorts on the basis of the design or analysis.

Item 6: Assessment of outcome.

Item 7: Was follow-up long enough for outcomes to occur.

Item 8: Adequacy of follow up of cohorts.
